# Supplementary material for: The interactome of intact mitochondria by cross-linking mass spectrometry provides evidence for coexisting respiratory supercomplexes
Source: Mol Cell Proteomics. 2017 Dec 8;17(2):216–32. doi: 10.1074/mcp.RA117.000470 (PMC5795388; doi:10.1074/mcp.RA117.000470)
Supplement: Supplemental Data [file supp_RA117.000470_133922_0_supp_23978_fzffwf.zip › spectra_annotation/mito_DR_spectra_annotation/497-1-5-1-19-1.pdf]

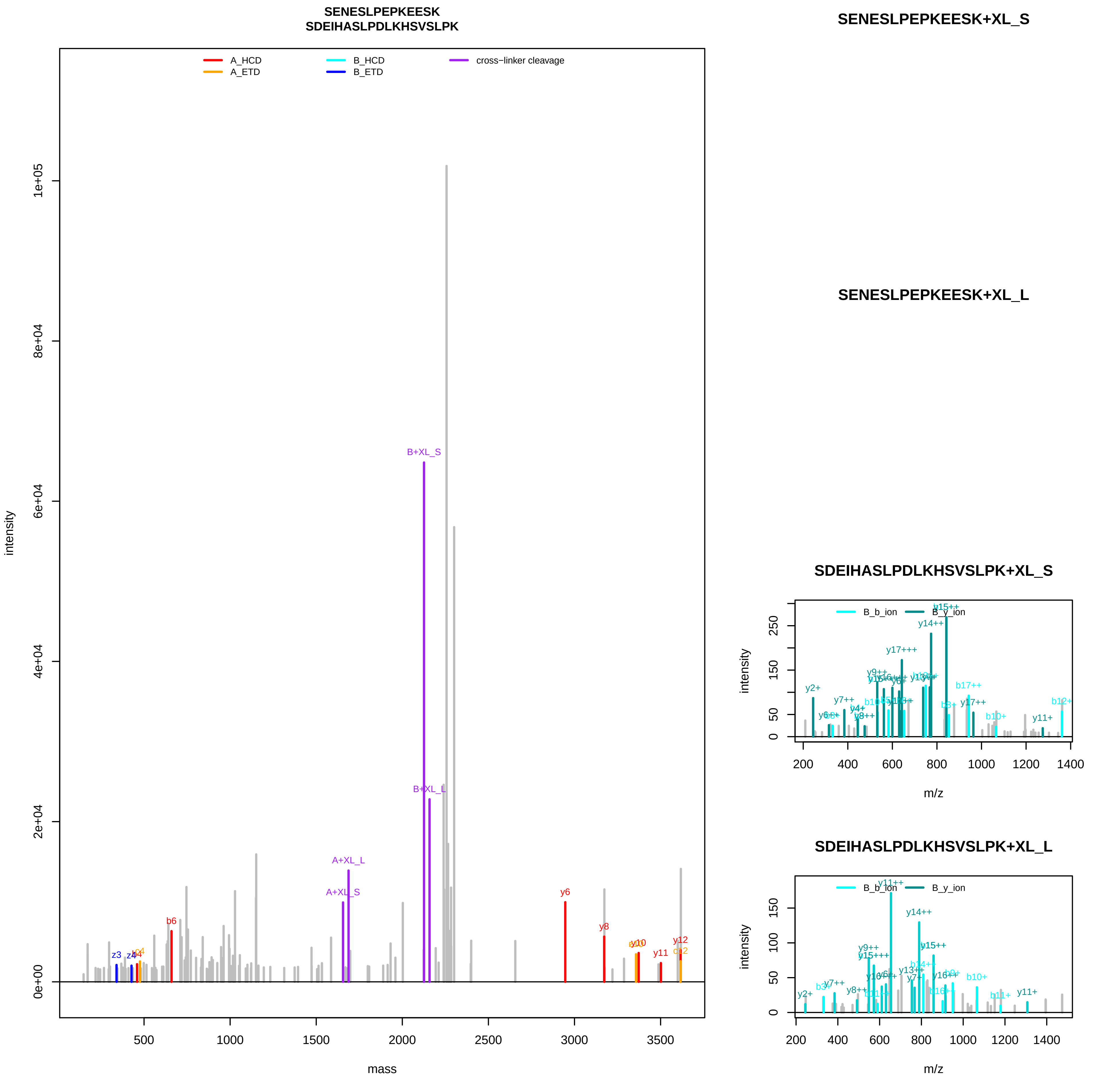
SENESLPEPKEESK
SDEIHASLPDLKHSVSLPK
A_HCD
A_ETD
B_HCD
B_ETD
cross−linker cleavage
intensity
mass
B+XL_S
B+XL_L
A+XL_L
A+XL_S
z3
z4
b6
y6
y8
y10
y11
y12
SENESLPEPKEESK+XL_S
SENESLPEPKEESK+XL_L
SDEIHASLPDLKHSVSLPK+XL_S
B_b_ion
B_y_ion
y15++
y14++
y17+++
y2+
y7++
y11+
b12+
b17++
y17++
m/z
SDEIHASLPDLKHSVSLPK+XL_L
y11++
y14++
y15++
y9++
y15+++
y13++
y16++
b10+
b3+
y7++
y2+
y8++
y11+
